# Modulation of Human Cardiac TRPM7 Current by Extracellular Acidic pH Depends upon Extracellular Concentrations of Divalent Cations

**DOI:** 10.1371/journal.pone.0170923

**Published:** 2017-01-27

**Authors:** Regina Mačianskienė, Mantė Almanaitytė, Aistė Jekabsone, Kanigula Mubagwa

**Affiliations:** 1 Institute of Cardiology, Lithuanian University of Health Sciences, Kaunas, Lithuania; 2 Neuroscience Institute, Lithuanian University of Health Sciences, Kaunas, Lithuania; 3 Department of Cardiovascular Sciences, University of Leuven, Leuven, Belgium; Cinvestav-IPN, MEXICO

## Abstract

TRPM7 channels participate in a variety of physiological/pathological processes. TRPM7 currents are modulated by protons but opposing effects of external pH (pH_o_) (potentiation *vs* inhibition) have been reported. TRPM7 has been less studied in human cardiomyocytes than in heart-derived non-cardiomyocyte cells. We used the whole-cell patch-clamp technique on isolated human atrial cardiomyocytes to investigate the impact of an acidic pH_o_ on the TRPM7 current. With voltage-dependent and other ion channels inhibited, cardiomyocytes were challenged with external acidification in either the presence or the absence of extracellular divalent cations. TRPM7 outward and inward currents were increased by acidic pH_o_ in extracellular medium containing Ca^2+^ and Mg^2+^, but suppressed by acidic pH_o_ in the absence of extracellular Ca^2+^ and Mg^2+^. The potentiating effect in the presence of extracellular divalents occurred at pH_o_ below 6 and was voltage-dependent. The inhibitory effect in the absence of extracellular divalents was already marked at pH_o_ of 6 and was practically voltage-independent. TRPM7 current density was higher in cardiomyocytes from patients with history of coronary vascular disease and the difference compared to cardiomyocytes from patients without history of myocardial ischemia increased with acidic pH_o_. We demonstrate that proton-induced modification of TRPM7 currents depends on the presence of extracellular Ca^2+^ and Mg^2+^. Variability of the TRPM7 current density in human cardiomyocytes is related to the clinical history, being higher in atrial fibrillation and in ischemic cardiomyopathy.

## Introduction

Mammalian cells express a diversity of transient receptor potential (TRP) channels, which underly a multitude of functions [1,2]. Among these channels, TRPM7 (transient receptor potential melastatin 7) appears to be ubiquitously expressed, with highest expression in tissues such as the heart [3,4]. In the last decade, molecular biology and immunodetection techniques have been used successfully to demonstrate the expression of TRPM7 at the gene and protein levels in the heart [5–7], including during embryonic development [8]. At the same time, however, the electrophysiological characterization of these or like channels in native cardiac cells has only involved very few studies [7,9–13]. This is due in particular to problems of separating currents carried by TRPM7 from those of different channels co-expressed in the same cell, for lack of specific inhibitors.

TRPM7 appears to be involved in many functions (for references see [14]), but the relationship between channel properties, activity or regulation and these functions remains largely unclear. Similarly, a growing number of studies has demonstrated an altered TRPM7 protein expression in a number of diseases, including hypertension [15] (for review see [16]), cancer [17,18], cerebral ischemia and stroke [19] (for review see [20]), and amyotrophic lateral sclerosis and Parkinson dementia [21] (see however [22]). In the heart, much of our understanding of the role of TRPM7 in cardiac pathophysiology has been obtained from studies involving heart-derived fibroblasts [10,23–25]. While the development of a TRPM7 knock-in mouse model has been used successfully to elucidate the role of TRPM7 channels in cellular and systemic response to Mg^2+^ deprivation [26], the recent development of a transgenic TRPM7 knock-out mouse model has helped evaluate the implication of these channels in impaired diastolic depolarization and automaticity [12] as well as in conduction defects [13]. An up-regulation of TRPM7 expression and its correlation with the severity of injury during myocardial ischemia/reperfusion have been shown in rat hearts [27].

We and others have demonstrated previously that TRPM7 currents could be measured in human atrial cardiomyocytes [7,11,28]. Interestingly, we found that the TRPM7 current could be already detectable immediately upon membrane patch break-in in freshly isolated human cardiomyocytes from atrial tissues of patients with sinus rhythm [11], as also observed by others in tissues from patients with atrial fibrillation [7]. This is in marked difference with findings in healthy ventricular cells of various animal species, in which the TRPM7 current could be induced only when a Mg^2+^-free pipette solution was used to dialyze the cells [9,29]. In addition, sensitivity to divalent cations was also shown to be enhanced in human atrial cardiomyocytes [11].

The role and regulation of TRPM7 in human cardiomyocytes remains unknown. Many factors prevailing under pathological conditions may influence the expression and activity of these channels. One such factor is pH, the intracellular and extracellular values of which may be modified under pathophysiological conditions. Although under normal conditions extracellular pH (pH_o_) is maintained at 7.4, under pathological conditions such as ischemia a dramatic reduction of pH_o_ can be obtained (see [30]). TRPM7 channels have been shown to be sensitive to extracellular and intracellular pH. While intracellular acidic solutions have been consistently shown to inhibit the channel (see [31]), the effects of pH_o_ have been variable, some studies showing stimulation, others demonstrating inhibition of channel activity. In the present study, we evaluated pH_o_ effects on TRPM7 in human atrial cardiomyocytes, with the aim to determine the influence of either the presence or the absence of divalent cations in the extracellular medium. The second aim was to determine whether pre-existing pathological conditions such as atrial fibrillation and/or ischemic heart disease could alter the properties or the expression of the TRPM7 current. Preliminary data have been presented as abstracts [32,33].

## Material and Methods

The study was carried out in accordance with the European Community guiding principles outlined in the Declaration of Helsinki, and was approved by the Ethics Committee of Biomedical Research of Kaunas Region, Lithuania (2014-05-23, Nr.BE-2-21).

### Cell isolation

We used cardiomyocytes obtained from small right atrial specimen removed from human hearts during cardiac surgery for coronary artery bypass or valve repair/replacement at the Hospital of the Lithuanian University of Health Sciences. Most of the patients were treated with Ca^2+^-channel blockers, angiotensin converting enzyme inhibitors, diuretics, which were stopped 24 h before surgery. In addition, all patients received anaesthesia and antibiotics. Written informed consent before surgical procedures was obtained. The patients’ characteristics are presented in Table 1.

**Table 1:** Clinical characteristics of the patients.

Patient data	SR	AF
Age range (years)	37–84	45–85
Mean age (years) ± SEM	64.7 ± 1.6	64.2 ± 3.5
Female, n (%)	37(37.0)	22(48.9)
Male, n (%)	63 (63.0)	23 (51.1)
Total, n (%)	100 (100)	45 (100)
**Surgical intervention**		
MV surgery, n (%)	5 (5.0)	12 (26.7)
AoV surgery, n (%)	14 (14.0)	6 (13.3)
AoV/MV surgery, n (%)	3 (3.0)	5 (11.1)
CABG surgery, n (%)	56 (56.0)	14 (31.1)
CABG and Valve surgery, n (%)	22 (21.0)	8 (17.8)
**Therapy, n (%)**		
Aspirine	33 (31.4)	15 (33.3)
Beta-blockers	51 (48.6)	24 (53.3)
Calcium antagonists	22 (21.0)	13 (28.9)
Angiotensin-converting enzyme (ACE) inhibitors	62 (59.1)	19 (42.2)
Antiarrhythmic agents	3 (2.9)	15 (33.3)
Diuretics	16 (15.2)	19 (42.2)
Nitrates	24 (22.9)	7 (15.6)
Medication not used	44 (41.9)	13 (28.9)

SR–sinus rhythm, AF–atrial fibrillation, MV–mitral valve, AoV–aortic valve, CABG–coronary artery bypass graft; Some patients underwent both valve and CABG surgery.

Atrial tissues were transported to the laboratory in cold (8–10°C) St. Thomas cardioplegic solution and were dissociated immediately as previously described [11]. In short, a small atrial specimen was fine-cut in an oxygenated nominally Ca^2+^-free Tyrode solution (see composition below) supplemented with 3 mg/mL 2,3-butanedione monoxime (BDM), which was washed out before enzyme application. The tissue chunks were transferred to a beaker with nominally Ca^2+^-free Tyrode solution supplemented with 1 mg/mL bovine serum albumin (BSA; Sigma; St. Louis, MO, USA), 1 mg/mL collagenase (215 U/mg, type 2; Worthington; Lakewood, NJ, USA), and 0.5 mg/mL proteinase (7–14 U/mg, type XXIV; Sigma) by continuous bubbling with 100% O_2_. After 30 min of shaking in a water bath at 37°C, the solution with both enzymes was replaced by fresh solution containing only collagenase (1 mg/mL), and shaken until cardiomyocytes appeared. When the yield appeared to be maximal, the leftover of tissue chunks were resuspended in a nominally Ca^2+^-free Tyrode solution and gently subjected to trituration by succion with a pipette. The cell suspension was filtered, centrifuged, and stored at room temperature in Tyrode solution with 0.18 mM Ca^2+^. Experiments were performed at room temperature (21 ± 2°C) on rod-shaped and Ca^2+^-tolerant cardiomyocytes.

### Electrophysiology

Experiments were performed using the VP-500 patch-clamp amplifier (Bio-Logic, Claix, France). Visual-Patch v1.30 software (Bio-Logic) was used to control all experimental parameters, cell stimulation, and current recordings. Symmetrical (4-s) voltage ramps from –120 mV to +80 mV and back to –120 mV were applied every 10 s from a holding potential of –80 mV. The ascending limb of the ramp allowed activation and inactivation of voltage-dependent Na^+^ and T-type Ca^2+^ channels. L-type Ca^2+^ currents were blocked by adding 10 μM nifedipine in all extracellular solutions. K^+^ currents were blocked by replacing K^+^ with Cs^+^. The TRPM7 currents were measured during the descending limb of the voltage ramp. Currents were not corrected for capacitive and leak components. They were filtered at 1 kHz and sampled at 5 kHz. The resistance of patch pipettes, pulled from glass capillaries with a horizontal puller (Sutter Instruments, Model P-97), was 1.5–2.2 MΩ. For averaging purposes, currents were normalized to the cell size and displayed as current densities (pA/pF). In all experiments extracellular solutions were applied using a rapid solution changer (RSC-200; Bio-Logic).

### Immunofluorescence

Enzymatically dissociated cardiomyocytes were allowed to settle on the bottom of 4-chamber slides for 15–20 minutes in normal Tyrode. Afterwards, the cells were covered with porcine collagen hydrogel coverslips (UAB Ferentis), fixed in 4% paraformaldehyde (PFA) for 15 min and then washed in physiologic buffer solution (PBS). Triton X-100 (0.1%) was used for 3 min permeabilisation. Non-specific binding of antibody was prevented by using blocking buffer containing 10% goat serum (Thermo Fisher Scientific, Waltham, MA, USA) in PBS for 1 hour. Cells were incubated with primary mouse monoclonal anti-TRPM7 ([S74-25] ab85016, Abcam Cambridge, UK) or rabbit polyclonal anti-TRPM7 antibody (ACC-047, Alamone labs, Jerusalem, Israel) diluted (1:200) in PBS containing 3% BSA in blocking buffer overnight at 4°C, and washed thereafter with PBS. For negative controls, incubation with primary antibody was omitted to check for non-specific binding of the secondary antibody. The cells were incubated for 1 hour with fluorescently-labelled secondary antibody (goat anti-mouse IgG (H+L) Alexa Fluor® 488 conjugate (A11029, Molecular probes, Eugene, OR, USA); dilution 1:200), co-stained with Phalloidin-Alexa Fluor® 546 (A22283, Molecular probes) dilution 1:100; for 20 min) and with Hoechst 33342 (B2261, Sigma Aldrich, St. Louis, MO, USA; 25 μg/mL; for 10 min) for labelling of the F-actin cytoskeleton and of the nucleus, respectively. Then, the hydrogels and media-chambers were removed and glass slides covered with ProLong Gold Anti-fade Reagent (P36934, Molecular probes) and coverslip glass, and sealed with clear nail polish. Cardiomyocytes were visualized under confocal laser scanning microscope (Olympus BX61, Hamburg, Germany) from which images were taken and the distribution of the fluorescence was analyzed using the Olympus Fluoview FV1000 and Image J softwares. Images are presented as stacks of 15–30 slices at maximum intensity.

### Solutions and drugs

The composition of the standard Tyrode solution used during cell isolation was (in mM): 135 NaCl, 5.4 KCl, 0–1.8 CaCl_2_, 0.9 MgCl_2_, 0.33 NaH_2_PO_4_, 10 glucose, and 10 HEPES (pH 7.4, adjusted with NaOH). During TRPM7 current measurements, cardiomyocytes were superfused with external solution of similar composition except that K^+^ was replaced by Cs^+^. Extracellular nominally divalent-free solutions were prepared by simply omitting Ca^2+^ and Mg^2+^ cations from the Tyrode solution. For the acidic external solutions with pH 5 and pH 4, we used 2-(N-morpholino)ethanesulfonic acid (MES) instead of HEPES as proton buffer. Standard internal solution contained (in mM): 130 Cs glutamate, 25 CsCl, 5 Na_2_ATP, 5.5 MgCl_2_ (0.7 mM free [Mg^2+^]_i_), 1 EGTA, 0.1 Na_2_GTP, 5 HEPES (pH 7.25; adjusted with CsOH). Free [Mg^2+^]_i_ was calculated with the CaBuf software (http://jgp.rupress.org/content/146/1/51/suppl/DC1). All chemicals were purchased from Sigma-Aldrich (St. Louis, MO). Zero-mM [Mg^2+^]_i_ (Mg^2+^-free) internal solution, used to up-regulate TRPM7 current, was prepared by omitting MgCl_2_ and by replacing 1 EGTA with 10 EDTA.

### Data analysis

Average data are presented as mean ± standard error of the mean (SEM), with n_c/p_ indicating the number of cells/patients studied under each experimental condition. Means were compared using the two-tailed *t*-test or ANOVA for evaluating differences between two groups or between multiple groups, respectively. P <0.05 was taken as threshold for statistical significance.

## Results

**Fig 1:**
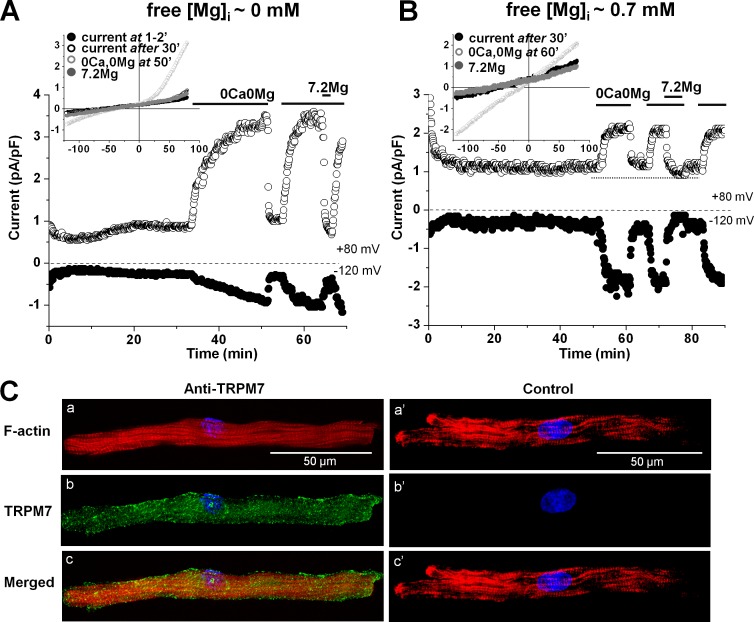
Detection of TRPM7 in human atrial cardiomyocytes. (A-B) Time diaries of whole-cell TRPM7 currents measured at +80 mV and –120 mV in cells dialyzed with either Mg^2+^-free internal solution (A) or with 0.7 mM [Mg^2+^]_i_ (B). Periods of exposure to 7.2 mM Mg^2+^ and nominally divalent-free extracellular solutions are indicated by horizontal bars. Insets: Traces from the same cells showing current voltage-relations obtained using voltage ramps from +80 mV to –120 mV. (C) Images of labeled TRPM7 proteins. Atrial cardiomyocytes were incubated with (a, b, c) or without (negative control; a’, b’, c’) TRPM7 primary antibody, and co-stained with Hoechst 33342 (for the nucleus; *blue*), Phalloidin-Alexa Fluor 546 (for F-actin cytoskeleton; *red*), goat anti-mouse Alexa Fluor 488 (for TRPM7; *green*). Uppermost row: image of F-actin staining. Middle row: image of TRPM staining. Lowermost row: merged image.

TRPM7 channels are known to be inhibited by intracellular Mg^2+^, hence their electrophysiological identification usually involves dialyzing cells with low free Mg^2+^ concentrations ([Mg^2+^]_i_). Fig 1A shows the time evolution of whole-cell currents measured at +80 mV and –120 mV while dialyzing with 0-mM [Mg^2+^]_i_ pipette solution, in either the presence or the absence of extracellular divalent cations. Both outward and inward currents increased with time before the removal of extracellular divalents, and a steady-state was reached after 20 min. The currents in the presence of extracellular Ca^2+^ and Mg^2+^ were small, due to voltage-dependent permeation block by the extracellular divalents [9,34]. Omitting divalent cations from the Tyrode solution rapidly and reversibly enlarged the outward and inward currents, consistent with large monovalent cation current flows under these conditions [3,9,35,36]. High [Mg^2+^]_o_ (7.2 mM) decreased both outward and inward currents.

We have previously shown that while the TRPM7 current was detectable in ventricular cardiomyocytes from various species (e.g. pig, rat) only during cell dialysis with 0-mM [Mg^2+^]_i_ pipette solution [9], measurable TRPM7 current can be detected in freshly isolated human atrial cardiomyocytes even with physiological [Mg^2+^]_i_ [11]. Fig 1B shows the time evolution of whole-cell currents using essentially the same voltage and extracellular solution change protocol as in Fig 1A, while dialyzing the cell with Mg^2+^-containing intracellular solution. [Mg^2+^]_i_ was set at 0.7 mM, a concentration corresponding to the physiological level. In this case, both inward and outward currents remained small, the current-voltage relationship became linear (probably reflecting a block of outward currents by Mg^2+^ entering the channels from the intracellular side), and there was no detectable time-dependent change in current amplitude during the 40–50 min cell dialysis in the presence of extracellular divalent cations. Removal of extracellular divalent cations increased the outward and inward currents. The changes were reversible upon readmission of extracellular divalent. As was the case during dialysis with Mg^2+^-free internal solution, the currents were sensitive to extracellular Mg^2+^, and application of 7.2 mM [Mg^2+^]_o_ actually decreased both outward and inward currents to levels lower than the initial baseline before removal of divalents (see dotted line in Fig 1B;), suggesting a contribution of TRPM7 to total membrane currents in human cardiomyocytes under physiological conditions.

TRPM7 protein expression in human atrial cardiomyocytes was confirmed by immunostaining. In Fig 1C the cells were viewed under confocal laser scanning microscopy. The left panels show images of one cell co-stained for nucleus (*blue*), F-actin cytoskeleton (*red*), and TRPM7 (*green*), whereas the right panels show similar images of another cardiomyocyte processed in conditions similar to those of the left panels except that no TRPM7 primary antibody was added in the incubation medium (negative control). The images demonstrate that TRPM7 is inhomogenously expressed in the plasma membrane of human atrial cardiomyocytes, with higher expression probably at lateral or end-to-end inter-connections between atrial cardiomyocytes. Similar preferential expression at points of interconnections was obtained using antibodies from different sources (see S1 and S2 Figs) and has also been observed in ventricular cells of various species [37].

### Opposite effects of extracellular acidic pH in the presence *vs* in the absence of divalent cations

As mentioned in the Introduction either an increase or a decrease of TRPM7 current by extracellular acidification have been observed. We were interested in elucidating the influence of divalent cations on the effect of extracellular pH (pH_o_).

**Fig 2:**
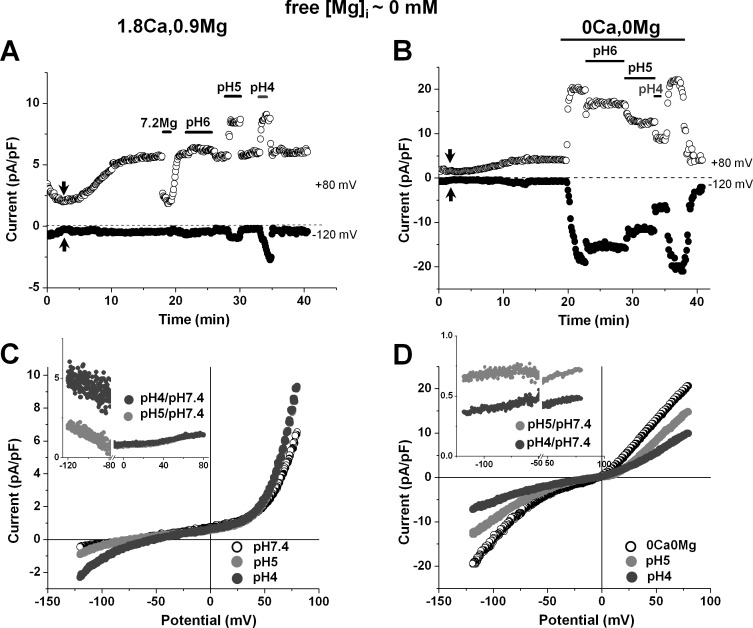
Effect of extracellular pH on the TRPM7 current in physiological and nominally divalent-free medium. (A-B) Currents recorded at +80 mV and –120 mV from two different human atrial cardiomyocytes before and during acidification of extracellular solutions either in the presence (A) or in the absence of extracellular divalent cations (B). Periods of exposure to acidic pH and nominally divalent-free solutions are indicated by horizontal bars. (C-D) Current-voltage relationships under steady-state conditions obtained using voltage ramps from +80 mV to –120 mV in the same cells as in A and B, respectively. (Insets in C-D) Ratios between TRPM7 currents recorded under various pH_o_ conditions over the current at pH 7.4. Breaks were made in the graph for potentials were currents at pH 7.4 were close to 0, making the ratio inaccurate. Notice that both the inward and outward currents are potentiated by acidic pH when perfusing with extracellular solution containing divalent cations, whereas they were reduced in nominally divalent-free medium.

Fig 2A and 2C illustrate the effect of changing pH_o_ on whole-cell TRPM7 current in a human atrial cardiomyocyte dialyzed with [Mg^2+^]_i_-free internal solution and superfused with extracellular solutions containing Ca^2+^ and Mg^2+^. Substantial [Mg^2+^]_o_-sensitve outward TRPM7 current developed with time, and a steady state was reached after 15–20 min (see also Fig 1A). Lowering pH_o_ to 6 caused only minor changes in the currents measured at +80 mV and at –120 mV. Further acidification to pH_o_ 5 or pH_o_ 4 caused marked increases in these currents. The effects were reversible upon return to physiological pH_o_, and reproducible upon renewed acidification. Corresponding current-voltage relations are displayed in Fig 2C. It is to be noticed that although the current amplitude changes in outward currents was larger in absolute values compared to the changes of inward currents, the relative increase of inward currents (when compared to the level at physiological pH_o_) was by far more pronounced. The ratio of currents at low pH_o_ over the current at pH_o_ of 7.4 steeply increased at negative potentials (see inset of Fig 2C), suggesting that protons exert a voltage-dependent effect on the TRPM7 current in the presence of extracellular divalent cations.

An experimental protocol similar to the one described in the above paragraph was applied while superfusing cells without extracellular divalent cations. In Fig 2B and 2D, after the unmasking of the TRPM7 current had reached steady-state in the presence of extracellular Ca^2+^ and Mg^2+^, these divalent cations were first removed and thereafter pH_o_ was modified in the continued absence of the divalents. As expected, the removal of extracellular divalent enlarged both inward and outward currents (see also Fig 1A). Under such experimental conditions, in contrast to the results obtained in the presence of divalents, subsequent extracellular acidification induced a pH_o_-dependent suppression of the TRPM7 currents. Superimposed current-voltage relationships at physiological and acidic pH_o_ in nominally divalent-free medium are displayed in Fig 2D. As illustrated in the inset of Fig 2D, the relative change in outward and inward current was of similar magnitude at positive and negative potentials, consistent with a practically voltage-independent (or only slightly voltage-dependent) effect of protons on the TRPM7 current in the absence of extracellular divalent cations, as also reported in our previous study in pig ventricular myocytes [9].

**Fig 3:**
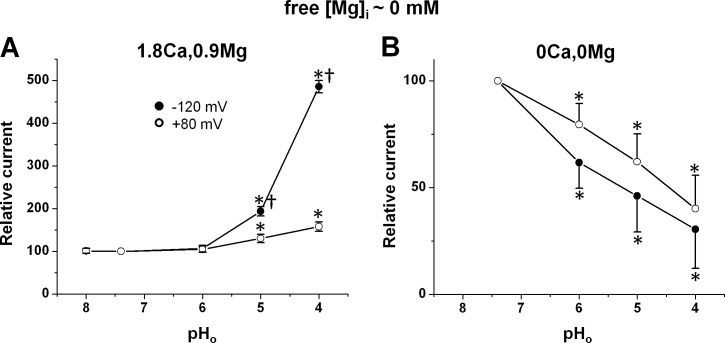
pH_o_ -dependent potentiation and inhibition of the TRPM7 current in human atrial cardiomyocytes. (A) Pooled data of the effects of extracellular acidification when perfusing with physiological solution containing divalent cations. (B) Pooled data of the effects when perfusing with extracellular divalent-free medium. All cells in (A) and (B) were dialysed with [Mg]_i_-free internal solution. Currents at positive (+80 mV; unfilled circles) and negative (-120 mV; filled circles) potentials are expressed relative to those measured pH_o_ 7.4 (taken as 100%). Each data point represents the mean ± SEM from 5 to 26 cells. Significant differences are denoted as *P <0.05 *vs* pH 7.4 for the same potential. ^†^P <0.05 for effect at –120 mV *vs* effect at -120 mV for the same pH_o_. Notice different scales in A *vs* B.

Such contrasting effects of acidic pH_o_ (voltage-dependent increase *vs* nearly voltage-independent decrease) in the presence *vs* in the absence of extracellular divalent cations were consinstently obtained on the TRPM7 current in human atrial cardiomyocytes in the present study. Fig 3 shows the mean relative changes in membrane currents induced at +80 mV and at -120 mV by the various pH_o_ levels in the presence of extracellular divalent cations (Fig 3A; n_c/p_ = 26/18) or in the absence of the divalents cations (Fig 3B; n_c/p_ = 19/14). These data clearly show that, in the presence of extracellular divalent cations, the magnitude of the TRPM7 current at both positive and negative potentials was only slightly modified with pH_o_ 6.0 (from 4.5 ± 0.32 to 4.6 ± 0.35 pA/pF, and from –0.5 ± 0.02 to –0.5 ± 0.04 pA/pF, at +80 mV and at –120 mV, respectively; n_c/p_ = 26/18), and that marked changes were obtained only at lower pH_o_. At these low pH_o_ there was a large difference in relative increases caused at +80 mV *vs* at –120 mV. In the presence of extracellular divalent the TRPM7 current at +80 mV was increased 1.37 fold (to 6.2 ± 0.60 pA/pF; n_c/p_ = 18/13) at pH_o_ 5 and 1.62 fold (to 7.4 ± 0.80 pA/pF; n_c/p_ = 13/10) at pH_o_ 4. Under the same conditions the inward current at –120 mV was increased 1.94 fold (to -0.9 ± 0.10 pA/pF) at pH_o_ 5 and 4.85 fold (to –2.3 ± 0.34 pA/pF) at pH_o_ 4. In contrast, in the absence of extracellular divalent cations, acidification-induced changes were already well marked at pH_o_ 6.0. In addition, there was no statistically significant difference in relative decreases at +80 mV *vs* at –120 mV caused by a given pH_o_ level. The TRPM7 current at +80 mV (18.9 ± 1.79 pA/pF) was decreased to 79% (15.1 ± 1.58 pA/pF; n_c/p_ = 17/12) at pH_o_ 6, to 62% (11.8 ± 1.54 pA/pF; n_c/p_ = 11/9) at pH_o_ 5, and to 40% (7.6 ± 1.19 pA/pF; n_c/p_ = 5/4) at pH_o_ 4. Under the same conditions the inward current measured at –120 mV (-17.9 ± 2.04 pA/pF at pH_o_ 7.4) was reduced to 62% (–11.1 ± 1.30 pA/pF; n_c/p_ = 17/12) at pH_o_ 6, to 46% (–8.3 ± 1.4 pA/pF; n_c/p_ = 11/9) at pH_o_ 5, and to 31% (–5.5 ± 1.0 pA/pF; n_c/p_ = 5/4) at pH_o_ 4.

The above data confirm different orders of magnitudes for the relative changes by a given pH_o_ of outward *vs* inward TRPM7 currents in the presence of divalent cations, and similar orders of magnitude changes of outward *vs* inward TRPM7 currents in nominally divalent-free solutions. They also suggest higher sensitivity to pH_o_ in the absence of extracellular divalent cations. We also tested the effect of extracellular alkalinizing to pH_o_ 8.0 (not illustrated). No significant modification of TRPM7 current was obtained under these experimental conditions (current change from 5.5 ± 0.28 pA/pF to 5.6 ± 0.34 pA/pF at +80 mV, and from –0.5 ± 0.07 pA/pF to -0.7 ± 0.05 pA/pF at –120 mV; P >0.05 in both cases; n_c/p_ = 3/2).

### Effect of acidic pH_o_ on the TRPM7 current under physiological conditions

**Fig 4:**
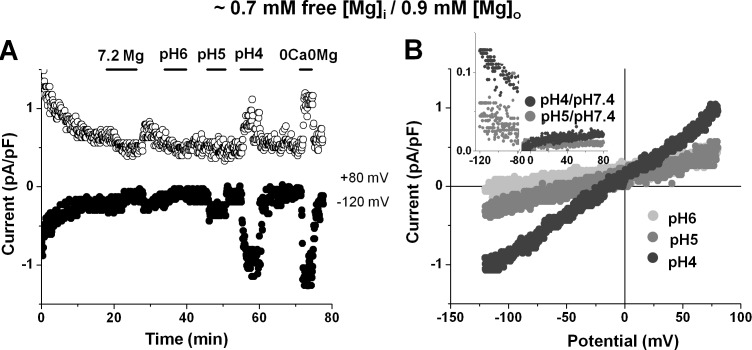
Effect of acidic external pH on the TRPM7 current in human cardiomyocyte under physiological conditions. (A-B) Time diaries of whole-cell currents measured at +80 mV and -120 mV (A) and current-voltage relationships (B) in the same cell, when dialyzed with internal solution containing 5.5 mM MgCl_2_ (calculated free [Mg^2+^]_i_ ~0.7 mM) and perfused initially with 1.8-mM [Ca^2+^]_o_, 0.9-mM [Mg^2+^]_o_ extracellular solutions at pH 7.4 and thereafter at pH 6.0, pH 5.0, and pH 4.0. Periods of exposure to 7.2 mM Mg^2+^, to acidic pH_o_, and to nominally divalent-free solutions are indicated by horizontal bars.

All the results on the effect of pH_o_ presented above were from cells dialyzed with practically 0 mM [Mg^2+^]_i_ to fully unmask TRPM7. Since TRPM7 can be active in atrial cardiac myocytes even in the presence of physiological [Mg^2+^]_i_ (see Fig 1B), we also tested whether the effect of acidic pH_o_ under such conditions (0.7 mM [Mg^2+^]_i_, 0.9 mM [Mg^2+^]_o_, 1.8 mM [Ca^2+^]_o_). Fig 4A shows the time diaries of currents measured at +80 mV and –120 mV, alongside with the corresponding current-voltage relationships (Fig 4B). As in Fig 1B, the small steady-state currents recorded at physiological pH (7.4) could be reversibly reduced by high [Mg^2+^]_o_, indicating they were due to TRPM7. Acidification of the extracellular medium also enhanced TRPM7 currents under such conditions. As expected, at pH_o_ of 6.0 no obvious changes could be detected compared to the currents at pH_o_ of 7.4. Further decreasing the pH_o_ to 5.0 and 4.0 potentiated the inward current amplitude (at –120 mV: from –0.3 ± 0.02 pA/pF to –0.6 ± 0.09 pA/pF (n_c/p_ = 8/7) and to -1.0 ± 0.39 pA/pF (n_c/p_ = 4/3), respectively; P <0.05 in both cases).

### Variability of the TRPM7 current in human atrial cardiomyocytes. Influence of underlying pathology

Since the cells used in the present study were obtained from tissues excised during routine cardiac surgery from patients with different pathologies, they offer a possibility to test whether the magnitude, properties or regulation of TRPM7 channels are affected by the underlying clinical conditions. We were also interested in examining the influence of the underlying diseases on the response to pH_o_.

**Fig 5:**
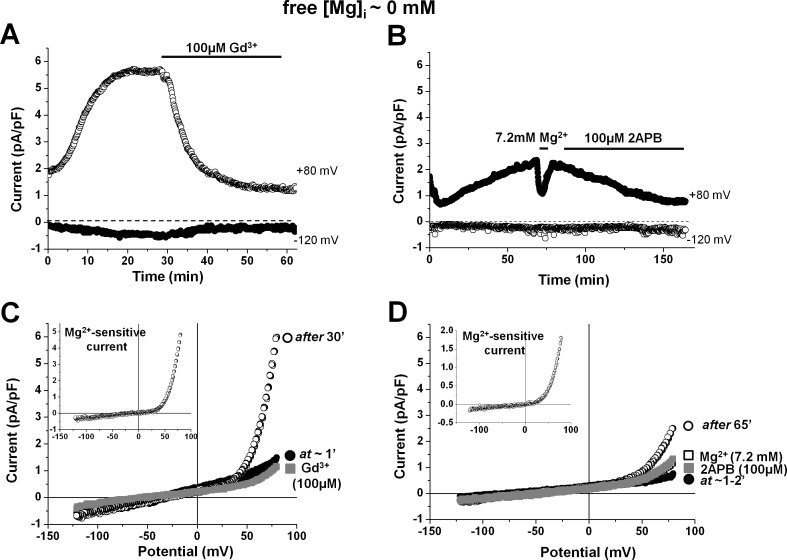
Variability of the TRPM7 current in human atrial cardiomyocytes from patients with AF. (A-B) Traces of the TRPM7 currents measured at +80 mV and –120 mV from two different human atrial cardiomyocytes, one from a patient with (A), the other from a patient without (B) history of myocardial ischemia, respectively. (C-D) Steady-state current-voltage relations obtained using voltage ramps from +80 mV to –120 mV in the same cells as in (A-B), respectively. (Insets C-D): Current-voltage relations of the typical Mg^2+^-sensitive current displaying steep outward rectification and *E*_rev_ ~0 mV. Periods of block of TRPM7 current with 7.2 mM Mg^2+^, 100 μM Gd^3+^ or 100 μM 2-APB are indicated by horizontal bars.

Our earlier study on human atrial cardiomyocytes indicated that there is a large variability of the TRPM7 current density in patients in sinus rhythm (SR) [11]. In the present study, we checked whether there is a similar variability in cardiomyocytes obtained from patients with atrial fibrillation (AF). Fig 5A and 5B illustrates the development of the TRPM7 current in two different human atrial cardiomyocytes during intracellular dialysis with [Mg^2+^]_i_-free solutions in the presence of extracellular divalent cations. Both cells were obtained from AF patients: one with (Fig 5A), and the other without (Fig 5B) documented history of coronary artery disease. In both cases outward currents developed progressively during washout of intracellular Mg^2+^. The developed current was typical of TRPM7: the current-voltage relation showed marked outward-going rectification (see insets in Fig 5C and 5D), due to large outward monovalent cation fluxes in the presence of only small inward divalent cation fluxes [9,38], was sensitive to block by high [Mg^2+^]_o,_ and the Mg^2+^-sensitive current had a reversal potential close to zero. The current was also sensitive to block by 100 μM Gd^3+^ or by 100 μM 2-APB. The current density at cell membrane rupture and at steady-state was of larger magnitude and transition to steady-state occurred faster in the cardiomyocyte derived from the ischemic cardiomypathy heart. Thus, TRPM7 current amplitude varied among different atrial cardiomyocytes from patients with AF, as was previously found in cardiomyocytes derived from patients in SR.

Given that the two cells presented above were from patients with and without history of myocardial ischemia, we examined whether ischemic cardiomyopathy has any influence on TRPM7 current amplitude. Mean values of the currents measured from all cells are presented in Table 2 and are grouped according to whether cells were obtained from patients with SR or AF, and subgrouped according to whether the patients presented ischemic cardiompyopathy or not. TRPM7 current density both at cell membrane rupture and at steady-state were larger in cardiomyocytes obtained from patients with AF, compared to those with SR. In every group, the currents were larger in cells obtained from hearts of patients with a history of myocardial ischemia. In addition, the kinetics of TRPM7 current unmasking during cell dialysis with 0 [Mg^2+^]_i_ was also different. The time to reach 50% of steady-state amplitude was 5.7 ± 0.26 min (n_c/p_ = 145/80) *vs* 8.5 ± 0.86 min (n_c/p_ = 52/20; P <0.0001), in cells derived from SR patients with *vs* without history of ischemia, respectively. These data suggest that variability in current density, hence in channel expression or activity, depends in part on myocardial remodeling and/or treatments associated with disease conditions such as AF and ischemic heart disease.

**Table 2:** Influence of underlying disease on amplitudes of the measured currents.

Current (pA/pF)	SR (n_c/p_ = 197/100)		AF (n_c/p_ = 94/45)	
	Not Isch.	Ischemic	Not Isch.	Ischemic
	(n_c/p_ = 52/20)	(n_c/p_ = 145/80)	(n_c/p_ = 39/19)	(n_c/p_ = 55/26)
*At cell rupture*				
+80 mV	1.4 ± 0.05	1.8 ± 0.04†	2.0 ± 0.09*	2.2 ± 0.08*
–120 mV	–0.2 ± 0.01	–0.3 ± 0.01†	–0.4 ± 0.02*	–0.4 ± 0.02*†
*Steady-state*				
+80 mV	3.5 ± 0.2	4.7 ± 0.13†	5.3 ± 0.32*	6.1 ± 0.27*†
–120 mV	–0.4 ± 0.01	–0.4 ± 0.01†	–0.5 ± 0.03*†	–0.6 ± 0.02*†

Densities of the currents (in pA/pF) recorded at positive (+80 mV) and negative (–120 mV) membrane potentials in human atrial cardiomyocytes: SR—cardiomyocytes from patients with sinus rhythm; AF—from patients with atrial fibrillation; Not Isch–from patients without ischemic injury (e.g. valve disease); Ischemic–from patients with history coronary artery disease. Each data point represents the mean ± SEM (n_c/p_ indicates number of cells *vs* patients).

Statistically significant differences (P <0.05) between AF and SR patient groups are denoted by *, and differences between ischemic and not ischemic cardiomyocytes are denoted by †.

**Fig 6:**
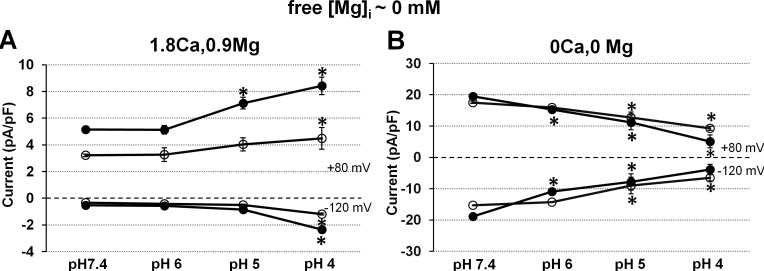
Comparison of the response to acidic pH_o_ between cardiomyocytes from patients with *vs* without previous myocardial ischemia. (A-B) Effects of acidic pH_o_ on TRPM7 current density measured in steady-state in extracellular solution either containing Ca^2+^ and Mg^2+^ (A) or nominally free of the divalent (B). Internal free-[Mg^2+^]_i_ = ~0 mM. Measurements taken at +80 mV and –120 mV. Each data point represents the mean ± SEM in cardiomyocytes obtained from patients with coronary artery disease (*filled symbols*), when superfusing with divalent cations (n_cells_ = 8–23) or without divalents (n_cells_ = 2–14), and in cells obtained from patients without history of coronary disease (*unfilled symbols)*, when superfusing with divalent cations (n_cells_ = 5–8), and without divalent cations (n_cells_ = 3–5). Statistically significant differences are denoted as *P <0.05 *vs* pH 7.4. Notice different scales in (A) *vs* (B).

Because of the larger TRPM7 current in cardiac myocytes of patients with AF, especially those with ischemic cardiomyopathy, any pathophysiological regulation of these channels by factors such as acidosis could also be more marked in the diseased hearts. We therefore examined the sensitivity to extracellular acidification in the cardiomyocytes from patients with *vs* without ischemic cardiomyopathy. Fig 6 shows the amplitudes of TRPM7 currents measured at +80 mV and at –120mV at different pH_o_ values. Currents at physiological pH_o_ were already larger in cells derived from ischemic cardiomyopathic hearts in consistence with results described above, and the difference increased further during acidification in the presence of extracellular divalent cations. However currents were of similar magnitude at all pH_o_ values in the absence of extracellular divalent cations.

## Discussion

Interest in TRPM7 channels has increased recently following the discovery that these channels participate in a variety of physiological/pathophysiological processes (see Introduction). However TRPM7 channel function varies depending on cell type [9,38,39]. In view of the scarcity of electrophysiological data on TRPM7 in myocardiac cells, and given the potential importance of these channels, we focussed our study on human atrial cells. Given the importance of pH changes under pathological conditions such as ischemia, and since opposite effects have been reported for the effect of extracellular acidosis, we sought to investigate the modulation of TRPM7 by acidic pH_o_ in human atrial cardiomyocytes. Our data demonstrate that both the inward and outward components of the TRPM7 current could be potentiated by an acidic pH_o_. Such proton-induced potentiation directly depends on presence of Ca^2+^ and Mg^2+^ in the extracellular medium, since the removal of these divalent cations caused a loss of the potentiation, replaced by the opposite effect, i.e. suppression of the TRPM7 current by extracellular acidification. Our data also suggest that the density of the TRPM7 current in human atrial cardiomyocytes highly depends on the underlying pathology, being higher in cells from patients with AF, especially those with history of coronary artery disease.

Earlier studies reported that proton-induced modulation on the TRPM7 channels is an important biophysical feature [40] (for review see [41]). Interestingly, using various cell lines [38,40,42–44] or human atrial fibroblasts [10], nearly all earlier studies consistently demonstrated strong potentiating effect only of the inward TRPM7 current, at an acidic pH_o_ set below 6.0. It was proposed that potentiating of inward TRPM7 current most likely is due to competition between protons and divalent cations for specific binding sites, and that binding of the protons increases selectivity of the channel to monovalent ion permeation. The increase of outward currents obtained in our experiments can also be explained by the same mechanisms as those involved in the increase of inward currents. Consistent with data presented here, using freshly isolated human atrial cardiomyocytes it has also been demonstrated by one research group [7] that TRPM7 current could be potentiated by low acidic pH_o_ at positive potentials as well. However, in that study, the effect was attributed to contaminating Cl^-^ currents, which can be present in human atrial cardiomyocytes and under certain circumstances could develop alongside with the TRPM7 current. In our study we paid special attention to identifying the typical biophysical properties of the TRPM7 current (see S3A and S3B Fig), in order not to include data contaminated with the Cl^-^ current. In case the developed current was a mixture of several currents, as demonstrated in S3C and S3D Fig, the experiment was excluded.

Our study provides a reconciliation of the apparently contradictory effects of extracellular acidification on the TRPM7 current. Divergent results in previous studies could be tentatively attributed to eventual species differences in the response to pH_o_. We show, using one cell type, that when extracellular divalent cations were absent an inhibitory instead of potentiation effect appears upon extracellular acidification. A similar regulation by acidic pH_o_ in nominally divalent-free extracellular medium was also found in experiments on pig ventricular cardiomyocytes or rat basophilic leukemia cells [9], but pH_o_ effects were not tested in the presence of extracellular divalent cations. Inhibitory effects of acidosis were also obtained on currents induced by extracellular divalent cation removal in neuronal cells (see [45]). All previous studies that reported a decrease of TRPM7 current by pH_o_ examined the effect in the absence of extracellular divalent but not in their presence.

Whereas the mechanism underlying pH_o_ effect in the presence of extracellular divalent can be accounted for by a competition between protons and the divalents for a binding site within the channel pore (see above), the mechanism underlying the pH_o_-induced decrease of TRPM7 current in the absence of extracellular divalent remains unclear. Given the reported suppressive effect of intracellular acidic pH on TRPM7 currents, it may be proposed that the effect of pH_o_ in the absence of extracellular divalent is due to permeation of protons and their subsequent binding to an intracellular inhibitory site or to a site within the pore but close to the intracellular mouth of the channel. Indeed, we previously noted that increased intracellular proton buffering capacity did prevent the inhibitory effect of extracellular acidification [9]. This hypothesis was not further tested in the present study. Although pH_o_ in the absence of divalent cations effects were largely voltage-insensitive, a slight tendency to increased inhibition by low pH_o_ was observed (compare data at –120 mV compared to +80 mV in Fig 3B; and see non-horizontal relationships in inset of Fig 2D). If protons were to inhibit after entering the cell via channels, some voltage-dependence may be an indirect result of larger proton influxes at larger driving forces. However since substantial inhibition was obtained even at potentials close or positive to the equilibrium potential for protons (E_H_; approximately +73 mV for pH_o_ 6), the hypothesis of inhibition via intracellular acidification has then to assume that protons also accumulated in the cell via other, eventually non-channel pathways, for example via the Na^+^-H^+^ exchanger.

To date, the functional role of TRPM7 is less clearly understood in heart cells compared to other cell types; e.g. neurons [19,46] or vascular smooth muscle cells (for review see [16]). Molecular and electrophysiological characterizations of TRPM7 in heart have focused predominantly upon cardiac fibroblasts, where contribution of these channels to cardiac remodeling, fibrosis and arrhythmogenesis has been proposed [10,25]. Atrial cardiomyocytes from a small group of AF patients (n = 3–5) were previously reported to express larger TRPM7 current [7]. Since our previous study demonstrated a large variability of the TRPM7 current magnitude among atrial samples from different human hearts with stable cardiac rhythm [11], we also sought to evaluate the current from larger groups of SR and AF patients. Our data, collected from 45 patients with AF and 100 patients with SR are in support of previous observations that the magnitude of the TRPM7 current density is higher in AF. Nevertheless, the TRPM7 current also displayed pronounced inter-individual variability in magnitude among cells from AF patients, as is the case for SR patients. Despite this variability, the current density varied less among cells from same human heart biopsy (not illustrated). This suggests that current variability may depend on the underlying disease and/or its associated treatment. In the present study, in support of our earlier preliminary observations [28,33] we found an increased TRPM7 current density in atrial cardiomyocytes from patients with ischemic coronary artery disease, independently of the presence of AF or SR. One aim of the study was also to test whether the underlying diseases has an influence on the sensitivity of TRPM7 to acidic pH_o_. The difference in TRPM7 current density between cells from patients with *vs* without history of ischemic coronary artery disease became larger at acidic pH_o_ (see Fig 6). However, the relative changes compared to the currents at pH 7.4 (increases by factors 1.7 *vs* 1.5 at pH_o_ 4 in the group with *vs* without history of ischemia) indicate that the current at pH 7.4 were scaled to the similar level, implying no major difference in the response to pH_o_ changes.

It is unlikely that the difference in TRPM7 current magnitude between atrial cardiomyocytes from patients with *vs* without history of myocardial ischemia can be explained by alterations in the TRPM7 biophysical properties. Despite the observed current density differences the typical characteristics of the TRPM7 currents (steep outward-going rectification in the presence of extracellular divalent cations, *E*_rev_ close to 0 mV, block by extracellular divalent cations, etc.) were preserved. A more likely explanation of the differences is a disease-related variation in the expression or activity levels of the channel. We noticed that whereas TRPM7 currents were larger in cells derived from ischemic cardiomyopathy hearts in the presence of extracellular divalents, no difference was found compared to cells from non-ischemic hearts in the absence of the divalents (Fig 6). The similarity in current density in extracellular nominally divalent free conditions indicates that there was no major change in channel expression. A recent report also indicates that TRPM7 protein expression is not modified in tissues from ischemic cardiomyopathy despite a down-regulation of the corresponding genes [47]. The mechanism underlying the difference in TRPM7 current density between ischemic cardiomyopathy and control in the presence of extracellular divalents remains unclear, but has therefore to be related to the activity of the TRPM7 channels. Since the increased TRPM7 current density in cardiomyocytes derived from ischemic hearts were also associated with a faster unmasking of TRPM7 current during cell dialysis with 0 [Mg^2+^]_i_, it is possible that the underlying disease conditions modified the sensitivity to inhibition by intracellular Mg^2+^, but this hypothesis will need more careful experimental testing. Also the hypotheis of a changed sensitivity to inhibition by extracellular divalents cannot be excluded, but a decreased sensitivity to extracellular divalent cations in unlikely to explain the difference between cells from ischemic and non-ischemic hearts, since in this case the differences would tend to decrease at acidic pH_o_. Finally, under normal conditions currents carried by TRPM7 are measured without contamination from TRPM6 due to lack of expression of the latter channel. It would be interesting to test whether TRPM6 expression is upregulated in ischemic cardiomyopathy, since by contaminating TRPM7 measurements or by forming heteromeres with TRPM7 it could confer changes in sensitivity to modulators such as divalent cations and protons.

One question raised by the findings of the present study is the role of TRPM7 in changes of resting and action potentials of cardiac cells under acidosis-inducing pathological conditions. Extracellular acidification has been shown to cause resting membrane depolarization and variable effects (either shortening or lengthening) on the action potential. The depolarization by pH_o_ has been largely attributed to an activation of Cl^-^ currents and could be suppressed by either dialyzing cells with low [Cl^-^]_i_ or by applying Cl^-^ channel blockers [48]. The contribution of TRPM7 remains unknown, especially since the manipulations to block Cl^-^ channels are nonspecific and could by themselves affect TRPM7 currents. Under conditions of acute ischemia pH_o_ decreases down to 6.0 (see [30]). In the present study such an acidic pH caused only minor changes in TRPM7 currents in the presence of extracellular divalents, suggesting that TRPM7 may not be a major contributor to the electrophysiological changes during acute ischemia in the heart, especially since the channel is also blocked by intracellular acidic pH and by the increased level of free Mg^2+^ following ischemia-associated ATP breakdown. However, a change in TRPM7 protein expression [27] and/or in channel modulation due to modified cellular signalling during acute ischemia may also affect the contribution the channels.

## Conclusions

The effect of an acidic pH_o_ on the TRPM7 current depends strongly on the presence or absence divalent cations in the extracellular milieu: potentiation by acidic pH_o_ in extracellular medium containing Ca^2+^ and Mg^2+^, inhibition in nominally divalent-free environment. TRPM7 current magnitude is variable among human atrial cardiomyocytes, and a large part of this variability may be caused by the underlying cardiac pathology.

## Limitations

Samples used in the present study came from inhomogeneous groups of patients. Even within a group such as that of patients with history of ischemic cardiomyopathy, differences in medications will exist. In addition the level of remodelling (e.g. compensatory hypertrophy) as well as co-morbidities due aging or other pathologies and inflammation may vary within a group, with possible influence on TRPM7 channel expression/modulation. Data from very large groups of patients may be needed before any observed change can be accurately linked with a given pathology.

## Supporting Information

S1 Fig Lack of effect of a removal of extracellular divalent cations on the immunodetected TRPM7 using a monoclonal antibody. Images of labeled TRPM7 proteins using a mouse monoclonal TRPM7 [S74-25] antibody obtained from Abcam (catalog number: ab85016). Atrial cardiomyocytes were first kept in the presence or absence of extracellular divalent cations for 2–12 hours, before being processed for immunostaining by incubating with TRPM7 primary antibody, and co-staining with Hoechst 33342 (for the nucleus; *blue*), Phalloidin-Alexa Fluor 546 (for F-actin cytoskeleton; *red*), goat anti-mouse Alexa Fluor 488 (for TRPM7; *green*). Leftmost image: F-actin staining. Middle image: TRPM staining. Rightmost image: mergedF-actin and TRPM7 stainings.
(TIF)

S2 Fig Lack of effect of a removal of extracellular divalent cations on the immunodetected TRPM7 using a polyclonal antibody. Images of labeled TRPM7 proteins using a rabbit polyclonal TRPM7 antibody from Alomone (catalog number: ACC-047). Atrial cardiomyocytes were first kept in the presence or absence of extracellular divalent cations for 2–12 hours, before being processed for immunostaining by incubating with TRPM7 primary antibody, and co-staining with Hoechst 33342 (for the nucleus; *blue*), Phalloidin-Alexa Fluor 546 (for F-actin cytoskeleton; *red*), goat anti-mouse Alexa Fluor 488 (for TRPM7; *green*). Leftmost image: F-actin staining. Middle image: TRPM staining. Rightmost image: merged F-actin and TRPM7 stainings.
(TIF)

S3 Fig Distinguishing features of typical TRPM7 current vs contaminating chloride current in human atrial cardiomyocytes. (S3A and S3C) Time diaries of whole-cell currents extracted at +80 mV and –120 mV in cells dialyzed with Mg^2+^-free internal solution. (S3B and S3D) Current-voltage relationships (CVR) using voltage ramps from +80 mV to –120 mV in the same cells as in S3A and S3C, respectively. In the insets (S3B and S3D): difference currents, obtained by subtracting the CVRs at cell membrane rupture from the CVRs after total currents had developed to new steady levels during cell dialysis. Notice the similar shape in time diaries (S3A *vs* S3C) but clear differences in CVR (S3B *vs* S3D; also compare the inserts). The following criteria were used to consider traces as contaminated by Cl^-^ currents: 1) the difference current had less marked outward-going rectification (with larger inward currents), and 2) *E*_rev_ was shifted to more negative potentials.
(TIF)

## References

[1] Ramsey I, Delling M, Clapham D. An introduction to TRP channels. Annu Rev Physiol 2006; 68: 619–647. 10.1146/annurev.physiol.68.040204.100431 16460286

[2] Venkatachalam K, Montell C. TRP channels. Annu Rev Biochem 2007; 76: 387–417. 10.1146/annurev.biochem.75.103004.142819 17579562 PMC4196875

[3] Nadler M, Hermosura M, Inabe K, Perraud A, Zhu Q, Stokes A, et al LTRPC7 is a Mg ATP-regulated divalent cation channel required for cell viability. Nature 2001; 411: 590–595. 10.1038/35079092 11385574

[4] Runnels L, Yue L, Clapham D. TRP-PLIK, a bifunctional protein with kinase and ion channel activities. Science 2001; 291: 1043–1047. 10.1126/science.1058519 11161216

[5] Kunert-Keil C, Bisping F, Krüger J, Brinkmeier H. Tissue-specific expression of TRP channel genes in the mouse and its variation in three different mouse strains. BMC Genomics 2006; 7: 159 10.1186/1471-2164-7-159 16787531 PMC1557673

[6] Fonfria E, Murdock P, Cusdin F, Benham C, Kelsell R, McNulty S. Tissue distribution profiles of the human TRPM cation channel family. J Recept Signal Transduct Res 2006; 26: 159–178. 10.1080/10799890600637506 16777713

[7] Zhang Y, Sun H, Chen K, Du X, Liu B, Chen L, et al Evidence for functional expression of TRPM7 channels in human atrial myocytes. Basic Res Cardiol 2012; 107: 282 10.1007/s00395-012-0282-4 22802050 PMC3442166

[8] Jin J, Desai B, Navarro B, Donovan A, Andrews N, Clapham D. Deletion of Trpm7 disrupts embryonic development and thymopoiesis without altering Mg2+ homeostasis. Science 2008; 322: 756–760. 10.1126/science.1163493 18974357 PMC2605283

[9] Gwanyana A, Amuzescu B, Zakharov S, Macianskiene R, Sipido K, Bolotina V, et al Magnesium-inhibited, TRPM6/7-like channel in cardiac myocytes: permeation of divalent cat ions and pH-mediated regulation. J Physiol (London) 2004; 559: 761–776. 15272039 10.1113/jphysiol.2004.067637 PMC1665187

[10] Du J, Jia X, Zheng Z, Hiroto T, Daniel F, David S, et al TRPM7-mediated Ca2+ signals confer fibrogenesis in human Atrial Fibrillation. Circ Res 2010; 106: 992–1003. 10.1161/CIRCRESAHA.109.206771 20075334 PMC2907241

[11] Macianskiene R, Martisiene I, Zablockaite D, Gendviliene V. Characterization of Mg2+ regulated TRPM7 like current in human atrial myocytes. J of Biomed Science 2012; 19: 75. 10.1186/1423-0127-19-75 PMC3431234 22891975

[12] Sah R, Mesirca P, Mason X, Gibson W, Bates-Withers C, Van den Boogert M, et al Timing of myocardial Trpm7 deletion during cardiogenesis variably disrupts adult ventricular function, conduction, and repolarization. Circulation 2013; 128: 101–114. 10.1161/CIRCULATIONAHA.112.000768 23734001 PMC3800036

[13] Sah R, Mesirca P, Van den Boogert M, Rosen J, Mably J, Mangoni M, et al Ion channel-kinase TRPM7 is required for maintaining cardiac automaticity. Proc Natl Acad Sci USA 2014; 110: E3037–3046. Erratum in: Proc Natl Acad Sci USA 2014;111(17):6528. 10.1073/pnas.1311865110 PMC3740880 23878236

[14] Penner R, Fleig A. The Mg2+ and Mg(2+)-nucleotide-regulated channel-kinase TRPM7. Handb Exp Pharmacol 2007; 179: 313–328. 10.1007/978-3-540-34891-7_19 PMC5663631 17217066

[15] Sontia B, Montezano A, Paravicini T, Tabet F, Touyz R. Down regulation of renal TRPM7 and increased inflammation and fibrosis in aldosterone-infused mice: effects of magnesium. Hypertension 2008; 51: 915–921. 10.1161/HYPERTENSIONAHA.107.100339 18268139

[16] Touyz R. Transient receptor potential melastatin 6 and 7 channels, magnesium transport, and vascular biology: implication in hypertension. Am J Physiol Heart Circ Physiol 2008; 294: H1103–H1118. 10.1152/ajpheart.00903.2007 18192217

[17] Kim B, Park E, Lee J, Jeon J, Kim S, So I. Suppression of transient receptor potential melastatin 7 channel induces cell death in gastric cancer. Cancer Sci 2008; 99: 2502–2509. Epub 2008 Nov 20. 10.1111/j.1349-7006.2008.00982.x 19032368 PMC11159291

[18] Guilbert A, Gautier M, Dhennin-Duthille I, Haren N, Sevestre H, Oudid-Ahidouch H. Evidence that TRPM7 is required for breast cancer cell proliferation. Am J Physiol Cell Physiol 2009; 297: C493–C502. Epub 2009 Jun 10. 10.1152/ajpcell.00624.2008 19515901

[19] Sun H, Jackson M, Martin L, Jansen K, Teves L, Cui H, et al Suppression of hipocampal TRPM7 protein prevents delayed neuronal death in brain ischemia. Nat Neurosci 2009; 12: 1300–1307. Epub 2009 Sep 6. 10.1038/nn.2395 19734892

[20] Bae C, Sun H. TRPM7 in cerebral ischemia and potential target for drug development in stroke. Acta Pharmacol Sin 2011; 32: 725–733. Epub 2011 May 9. 10.1038/aps.2011.60 21552293 PMC4009967

[21] Hermosura M, Nayakanti H, Dorovkov M, Calderon F, Ryazanov A, Haymer D, et al A TRPM7 variant shows altered sensitivity to magnesium that may contribute to the pathogenesis of two Guamanian neurodegenerative disorders. Proc Natl Acag Sci U S A 2005; 102: 11510–11515. 10.1073/pnas.0505149102 PMC1183597 16051700

[22] Hara K, Kokubo Y, Ishiura H, Fukuda Y, Miyashita A, Kuwano R, et al TRPM7 is not associated with amyotrophic lateral sclerosis parkinsonism dementia complex in the Kii peninsula of Japan. Am J Med Genet B Neuropsychiatr Genet 2010; 153B: 310–313. 10.1002/ajmg.b.30966 19405049

[23] Runnels L, Yue L, Clapham D. The TRPM7 channel is inactivated by PIP2hydrolysis. Nature Cell Biol 2002; 4. 10.1038/ncb781 11941371

[24] Pujadas S, Vidal-Perez R, Hidalgo A, Letaa R, Carreras F, Barros A, et al Correlation between myocardial fibrosis and the occurrence of atrial fibrillation in hypertrophic cardiomyopathy: A cardiac magnetic resonance imaging study. Eur J Radiol 2010; 75: e88–91. 10.1016/j.ejrad.2009.12.012 20079992

[25] Yu Y, Chen S, Xiao C, Jia Y, Guo J, Jiang J, et al TRPM7 is involved in angiotensin II induced cardiac fibrosis development by mediating calcium and magnesium influx. Cell Calcium 2014; 55: 252–260. 10.1016/j.ceca.2014.02.019 24680379

[26] Ryazanova L, Hu Z, Suzuki S, Chubanov V, Fleig A, Ryazanov A. Elucidating the role of the TRPM7 alpha-kinase: TRPM7 kinase inactivation leads to magnesium deprivation resistance phenotype in mice. Sci Rep 2014; 4: 7599 10.1038/srep07599 25534891 PMC4274504

[27] Demir T, Yumrutas O, Cengiz B, Demiryurek S, Unverdi H, Kaplan D, et al Evaluation of TRPM (transient receptor potential melastatin) genes expressions in myocardial ischemia and reperfusion. Mol Biol Rep 2014; 41: 2845–2849. Epub 2014 Jan 21. 10.1007/s11033-014-3139-0 24445530

[28] Martisiene I, Gendviliene V, Zablockaite D, Macianskiene R. Characterization of TRPM7-like current in human atrial cardiomyocytes. Available at: http://www.physoc.org/proceedings/abstract/Proc%20Physiol%20Soc%2023PC267 [Accessed July 14, 2011]. 2011. 10.1186/1423-0127-19-75 PMC3431234 22891975

[29] Macianskiene R, Gwanyanya A, Vereecke J, Mubagwa K. TRPM7-like Channel in Cardiac Myocytes by Nonhydrolysable GTP Analogs: Involvement of Phosphoinositide Metabolism. Cell Physiol Biochem 2008; 22: 109–118. 10.1159/000149788 18769037

[30] Carmeliet E. Cardiac ionic currents and acute ischemia from channels to arrhythmias. Physiol Rev 1999; 79: 917–1017. 10390520 10.1152/physrev.1999.79.3.917

[31] Chokshi R, Matsushita M, Kozak J. Detailed examination of Mg2+ and pH sensitivity of human TRPM7 channels. Am J Physiol Cell Physiol 2012; 302: C1004–C1011. Epub 2012 Feb 1. 10.1152/ajpcell.00422.2011 22301056 PMC3330740

[32] Almanaityte M, Martisiene I, Benetis R, Macianskiene R. The pH sensitivity of TRPM7-like current in human atrial cardiomyocytes. Acta Physiol (Abstracts) 2015; 115: 74–75. Available at: http://www.feps.org/yuklemeler/FEPS%20Kaunas%202015%202020Acta%202020Physiologica.pdf.

[33] Almanaityte M, Martisiene I, Gendviliene V, Macianskiene R. Upregulation of TRPM7-like current in ischemia damaged human atrial cardiomyocytes. Biophys J (Abstracts) 2014; 106: 753a Available at: http://www.sciencedirect.com/science/article/pii/S0006349513054052.

[34] Kerschbaum H, Kozak J, Cahalan M. Polyvalent cations as permeant probes of MIC and TRPM7 pores. Biophys J 2003; 84: 2293–2305. 10.1016/S0006-3495(03)75035-8 12668438 PMC1302796

[35] Mubagwa K, Stengl M, Flameng W. Extracellular divalent cations block a cation non-selective conductance unrelated to calcium channels in rat cardiac muscle. J Physiol (Lond) 1997; 502: 235–247. 9263906 10.1111/j.1469-7793.1997.235bk.x PMC1159545

[36] Zakharov S, Smani T, Leno E, Macianskiene R, Mubagwa K, Bolotina V. Monovalent cation (MC) current in cardiac and smooth muscle cells: regulation by intracellular Mg2+ and inhibition by polycations. Br J Pharmacol 2003; 138: 234–244. 10.1038/sj.bjp.0705074 12522095 PMC1573659

[37] Istrate B, Gwanyanya A, Driesen R, Bito V, Mubagwa K. Non-homogenous distribution of TRPM7 in cardiac ventricular myocytes. Available at:http://users.ugent.be/~jvdvoord/physiology&pharmacology/2012AutumnMeetingAbstractbook.pdf [Accessed October 26, 2012] 2012.

[38] Monteilh-Zoller M, Hermosura M, Nadler M, Scharenberg A, Penner R, Fleig A. TRPM7 provides an ion channel mechanism for cellular entry of trace metal ions. J Gen Physiol 2003; 121: 49–60. 10.1085/jgp.20028740 12508053 PMC2217320

[39] Chubanov V, Waldegger S, Mederos y Schnitzler M, Vitzthum H, Sassen M, Seyberth H, et al Disruption of TRPM6/TRPM7 complex formation by a mutation in the TRPM6 gene causes hypomagnesemia with secondary hypocalcemia. Proc Natl Acad Sci USA 2004; 101: 2894–2899. 10.1073/pnas.0305252101 14976260 PMC365716

[40] Numata T, Okada Y. Proton conductivity through the human TRPM7 channel and its molecular determinants. J Biol Chem 2008; 283: 15097–15103. 10.1074/jbc.M709261200 18390554 PMC3258882

[41] Fleig A V. C. TRPM7. Handb Exp Pharmacol 2014; 222: 521–546. Review. 10.1007/978-3-642-54215-2_21 24756720 PMC5663634

[42] Jiang J, Li M, Yue L. Potentiation of TRPM7 inward currents by protons. J Gen Physiol 2005; 126: 137–150. 10.1085/jgp.200409185 16009728 PMC2266571

[43] Li M, Jiang J, Yue L. Functional characterisation of homo- and heteromeric channel kinases TRPM6 and TRPM7. J Gen Physiol 2006; 127: 525–537. 10.1085/jgp.200609502 16636202 PMC2151519

[44] Li M, Du J, Jiang J, Ratzan W, Su L, Runnels L, et al Molecular determinants of Mg2+ and Ca2+ permeability and pH sensitivity in TRPM6 and TRPM7. J Biol Chem 2007; 208: 25817–25831. 10.1074/jbc.M608972200 PMC3239414 17599911

[45] Chu X, Zhu X, Wei W, Li G, Simon R, MacDonald J, et al Acidosis decreases low Ca2+-induced neuronal excitation by inhibiting the activity of calcium-sensing cation channels in cultured mouse hippocampal neurons. J Physiol (Lond) 2003; 550: 385–399. 12777448 10.1113/jphysiol.2003.043091 PMC2343034

[46] Krapivinsky G, Mochita S, Krapivinsky L, Cibulsky S, Clapham D. The TRPM7 ion channel function in cholinergic synaptic vesicles and affects transmitter release. Neuron 2006; 52: 485–496. 10.1016/j.neuron.2006.09.033 17088214

[47] Ortega A, Roselló-Lletí E, Tarazón E, Gil-Cayuela C, Lago F, González-Juanatey J-R, et al TRPM7 is down-regulated in both left atria and left ventricle of ischaemic cardiomyopathy patients and highly related to changes in ventricular function. ESC HEART FAILURE 2016; 3: 220–224. 10.1002/ehf2.12085 27818786 PMC5071679

[48] Komukai K, Brette F, CH O. Electrophysiological response of rat atrial myocytes to acidosis. Am J Physiol herat Circ Physiol 2002; 283: H715–H724. 10.1152/ajpheart.01000.2001 12124220

