# Supplementary figures and images for: Modulation of Human Cardiac TRPM7 Current by Extracellular Acidic pH Depends upon Extracellular Concentrations of Divalent Cations
Source: PLoS One. 2017 Jan 27;12(1):e0170923. doi: 10.1371/journal.pone.0170923 (PMC5271359; doi:10.1371/journal.pone.0170923)

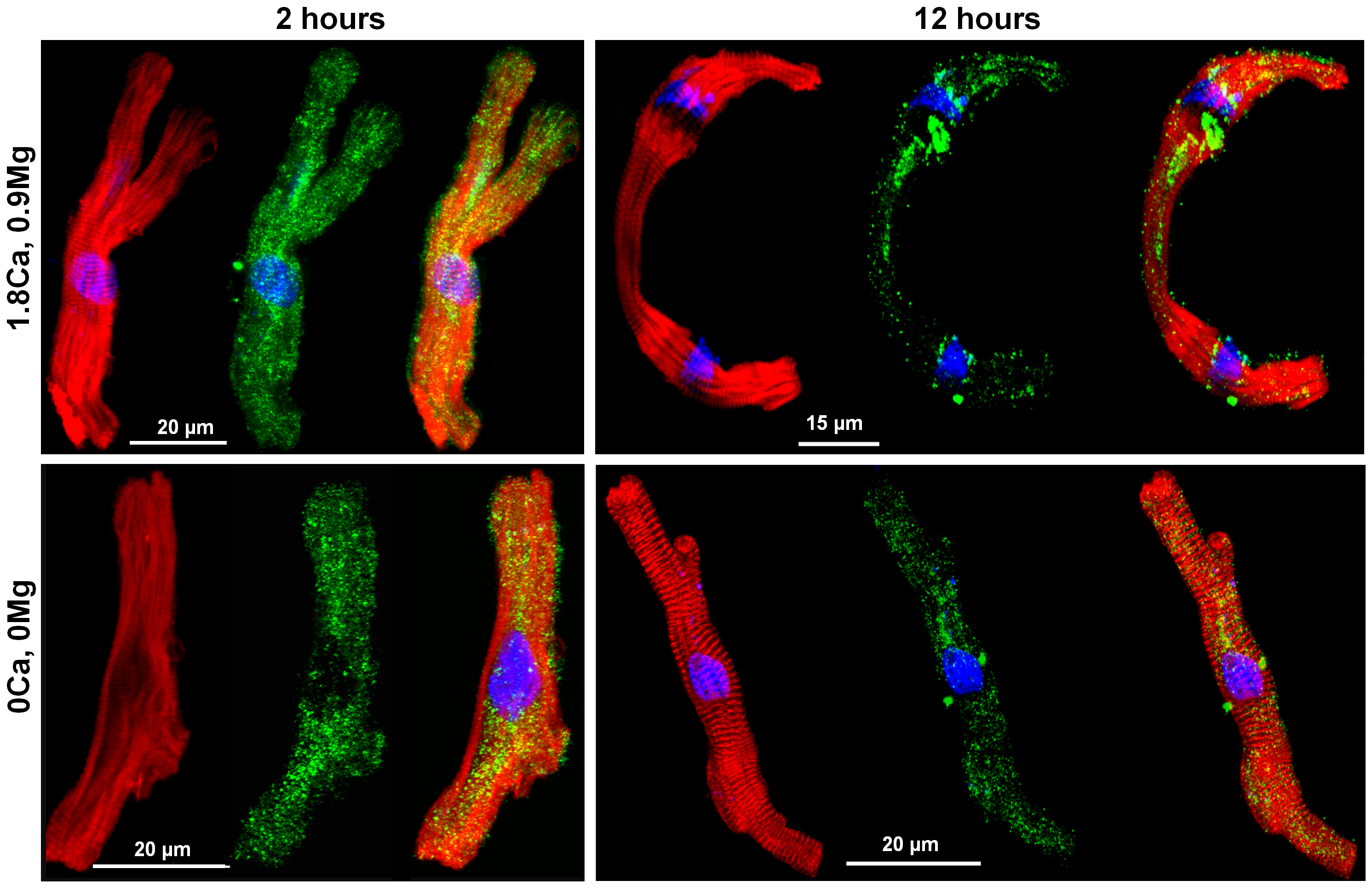

Supplement: S1 Fig — Images of labeled TRPM7 proteins using a mouse monoclonal TRPM7 [S74-25] antibody obtained from Abcam (catalog number: ab85016). Atrial cardiomyocytes were first kept in the presence or absence of extracellular divalent cations for 2–12 hours, before being processed for immunostaining by incubating with TRPM7 primary antibody, and co-staining with Hoechst 33342 (for the nucleus; blue), Phalloidin-Alexa Fluor 546 (for F-actin cytoskeleton; red), goat anti-mouse Alexa Fluor 488 (for TRPM7; green). Leftmost image: F-actin staining. Middle image: TRPM staining. Rightmost image: mergedF-actin and TRPM7 stainings. (TIF) [file pone.0170923.s001.tif]

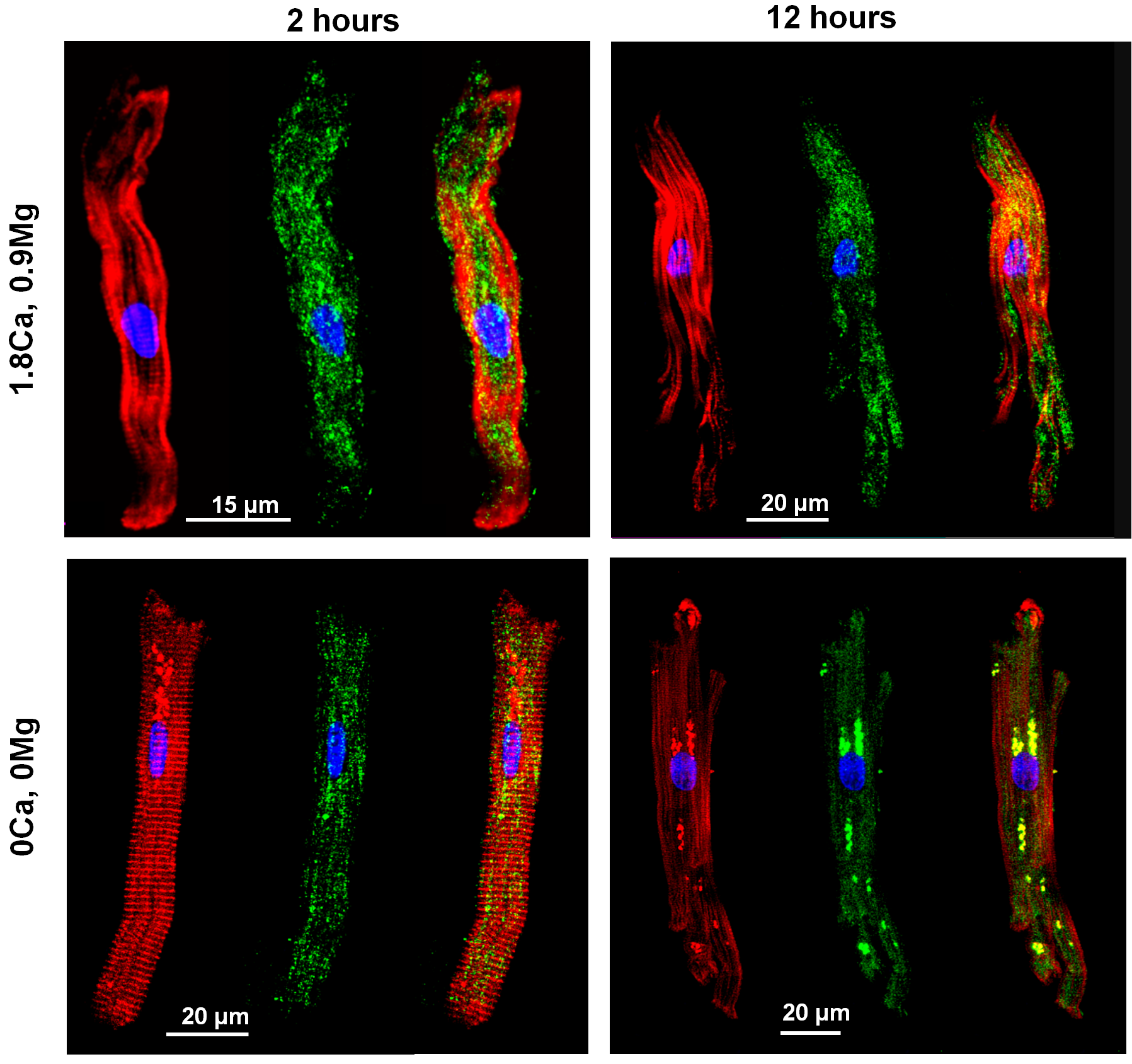

Supplement: S2 Fig — Images of labeled TRPM7 proteins using a rabbit polyclonal TRPM7 antibody from Alomone (catalog number: ACC-047). Atrial cardiomyocytes were first kept in the presence or absence of extracellular divalent cations for 2–12 hours, before being processed for immunostaining by incubating with TRPM7 primary antibody, and co-staining with Hoechst 33342 (for the nucleus; blue), Phalloidin-Alexa Fluor 546 (for F-actin cytoskeleton; red), goat anti-mouse Alexa Fluor 488 (for TRPM7; green). Leftmost image: F-actin staining. Middle image: TRPM staining. Rightmost image: merged F-actin and TRPM7 stainings. (TIF) [file pone.0170923.s002.tif]

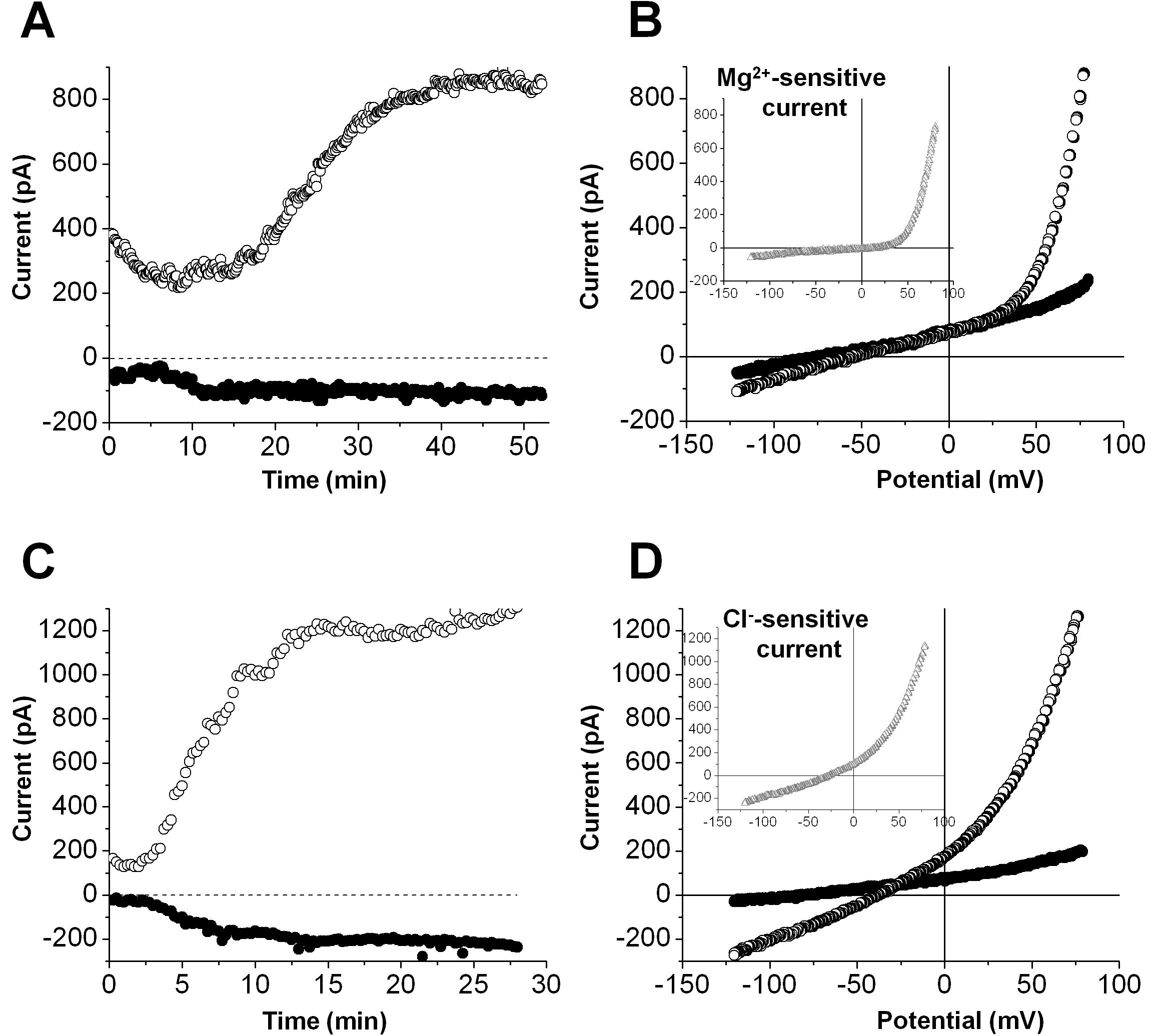

Supplement: S3 Fig — (S3A and S3C) Time diaries of whole-cell currents extracted at +80 mV and –120 mV in cells dialyzed with Mg2+-free internal solution. (S3B and S3D) Current-voltage relationships (CVR) using voltage ramps from +80 mV to –120 mV in the same cells as in S3A and S3C, respectively. In the insets (S3B and S3D): difference currents, obtained by subtracting the CVRs at cell membrane rupture from the CVRs after total currents had developed to new steady levels during cell dialysis. Notice the similar shape in time diaries (S3A vs S3C) but clear differences in CVR (S3B vs S3D; also compare the inserts). The following criteria were used to consider traces as contaminated by Cl- currents: 1) the difference current had less marked outward-going rectification (with larger inward currents), and 2) Erev was shifted to more negative potentials. (TIF) [file pone.0170923.s003.tif]
